# Sexual dimorphisms in skeletal muscle: current concepts and research horizons

**DOI:** 10.1152/japplphysiol.00529.2023

**Published:** 2024-05-23

**Authors:** Marianne E. Emmert, Andrew S. Emmert, Qingnian Goh, Roger Cornwall

**Affiliations:** ^1^Division of Orthopaedic Surgery, Cincinnati Children’s Hospital Medical Center, Cincinnati, Ohio, United States; ^2^Department of Orthopaedic Surgery, University of Cincinnati College of Medicine, Cincinnati, Ohio, United States; ^3^Division of Developmental Biology, Cincinnati Children's Hospital Medical Center, Cincinnati, Ohio, United States; ^4^Department of Pediatrics, University of Cincinnati College of Medicine, Cincinnati, Ohio, United States

**Keywords:** muscle biology, muscle physiology, sex as a biological variable, sexual dimorphisms, skeletal muscle

## Abstract

The complex compositional and functional nature of skeletal muscle makes this organ an essential topic of study for biomedical researchers and clinicians. An additional layer of complexity is added with the consideration of sex as a biological variable. Recent research advances have revealed sexual dimorphisms in developmental biology, muscle homeostasis, adaptive responses, and disorders relating to skeletal muscle. Many of the observed sex differences have hormonal and molecular mechanistic underpinnings, whereas others have yet to be elucidated. Future research is needed to investigate the mechanisms dictating sex-based differences in the various aspects of skeletal muscle. As such, it is necessary that skeletal muscle biologists ensure that both female and male subjects are represented in biomedical and clinical studies to facilitate the successful testing and development of therapeutics for all patients.

## Graphical abstract

**Figure:**
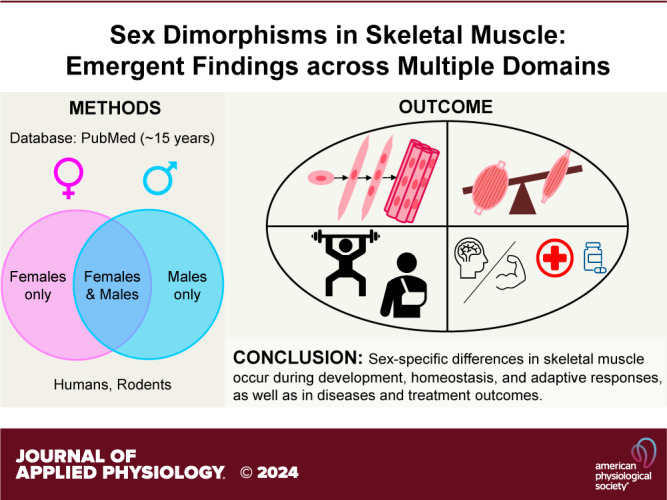

## INTRODUCTION

As the most abundant tissue in the human body, skeletal muscle is a highly studied organ for its structural and functional complexity (1). The physiology of skeletal muscle has been a topic of interest for researchers and clinicians seeking to improve treatment strategies for individuals living with skeletal muscle disorders. Early studies describing muscle composition and development believed that sex was a confounding variable in experimental models and, as a result, only included male subjects (2). However, contemporary research has identified a litany of observed sex differences in numerous aspects of skeletal muscle biology and physiology, arguing for the inclusion of sex as a variable in experimental models.

The National Institutes of Health (NIH) recently implemented a new policy recognizing sex as a biological variable (SABV) (3). This policy of SABV addresses the expectation that sex will be a factor in the design, analysis, and reporting of research studies involving vertebrate animals and humans (3). The inclusion of sex in research studies is crucial to the pillars of science: reproducibility, transparency, and rigor. Thus, the recognition of SABV by the NIH provides researchers with the impetus to fill the gaps in our current understanding of normal and disordered biological processes, such as those central to skeletal muscle. In this review, we will focus our attention on the most recent findings (within the last ∼15 years) of sexual dimorphisms in skeletal muscle development, muscle homeostasis, muscular remodeling, and disease along with the molecular, cellular, and metabolic mechanisms underlying these physiological differences. Where applicable, we will also examine the roles of the primary sex hormones, estrogen and testosterone, in mediating key myogenic processes and responses. This review is not meant to be exhaustive nor systematic. Indeed, the strength of evidence for sex differences in the different domains of skeletal muscle covered here varies greatly. Rather, the review will highlight select examples of sex dimorphisms documented in skeletal muscle biology to demonstrate the breadth of processes in which such dimorphisms may potentially occur, and to underscore the necessity to include sex as a factor in skeletal muscle research and beyond.

## SKELETAL MUSCLE DEVELOPMENT

Skeletal muscle development is a multifaceted embryonic process governed by a complex array of myogenic factors that regulate determination and differentiation of somites into progenitor cells, ultimately leading to the formation of muscle tissue (myogenesis) (4). At this nascent stage, sex differences are already present, highlighting a critical role of SABV in skeletal muscle independent of postnatal hormonal development and sexual maturation. In the following section, we will discuss current evidence of sexual dimorphisms with regards to embryonic myogenesis and postnatal growth (Table 1).

**Table 1.** Summary of reported sex differences in the domain of skeletal muscle development

Domain	Species	Female	Female and Male	Male
*Embryonic/in utero*				
•Baseline (5)	Bovine			↑ Number of muscle cells vs. female ↑ Muscle expression of myogenic, adipogenic, fibrogenic markers vs. female
•Maternal overnutrition (5)	Bovine	No interaction effect	↑ Muscle protein content vs. sex-matched controls ↑ Muscle expression of myogenic, adipogenic, fibrogenic markers vs. sex-matched controls	No interaction effect
•Maternal obesity (6)	Mouse	↑ RNA synthesis and transport pathways in fetal muscles	Genes expressed beyond the fetal period	↑ Metabolic pathways in fetal muscles
*Neonatal period*				
•Baseline (7–9)	Mouse			↑ Muscle mass, CSA, volume, protein content vs. female
•Mstn inhibition (8)	Mouse	Greater ↑ in muscle mass, volume, protein content vs. sex-matched controls		↑ Muscle mass, CSA, volume, protein content vs. sex-matched controls
•Sympathectomy (9)	Mouse	No effect on muscle growth vs. sex-matched controls		↓ CSA, volume vs. sex-matched controls
•β-Adrenergic stimulation (9)	Mouse, Rat	No effect on muscle growth vs. sex-matched controls		↑ CSA, volume vs. sex-matched controls
*Childhood*				
•Gonadectomy (7)	Mouse		↓ Myofiber CSA vs. sex-matched controls; restored with testosterone but not E2	
*Puberty*				
•Ovariectomy (10)	Mouse	↓ Myofiber CSA and grip strength vs. sex-matched controls; restored with E2		N/A
•Myofiber deletion of *Erβ* (11)	Mouse	↓ Muscle mass, myofiber CSA, grip strength vs. sex-matched controls		No effect on muscle growth and function vs. sex-matched controls
•Orchiectomy (12–14)	Mouse	N/A		↓ Muscle mass and myofiber CSA vs. sex-matched controls; restored with testosterone
•*Mstn* null mutation (15, 16)	Mouse	↑ Muscle mass of triceps vs. sex-matched controls (15) ↑ Myofiber CSA in soleus, plantaris, gastrocnemius vs. sex-matched controls (16)		Greater ↑ in muscle mass of triceps vs. sex-matched controls (15) ↑ Myofiber CSA in soleus vs. sex-matched controls (16)

CSA, cross-sectional area; E2, estradiol.

### Sexual Dimorphisms in Skeletal Muscle Embryonic Myogenesis

Embryonic skeletal muscle formation in vertebrates begins during the fourth week of development (4). At this time, specialized mesodermal cells (myoblasts) begin to mitotically divide, initiating myogenesis (17). By the ninth week of development, a sufficient number of myoblasts will proliferate, fuse, and ultimately differentiate into multinucleated skeletal muscle cells or muscle fibers. After 5 mo of development, myofilaments, or protein filaments with functional roles in muscle contraction, begin to form in the muscle fiber. During embryonic development, however, not all of the myoblast population differentiates. The myoblasts that do not fuse reside inside the developing external lamina of the muscle fiber as muscle satellite cells (18). Although quiescent under resting conditions, satellite cells play a role in regeneration and repair by proliferating and producing new muscle fibers during development or after postnatal injury (18). Expression of the transcription factor PAX7 is an indicator of quiescent satellite cells, whereas activated satellite cells coexpress PAX7 and MYOD (19). Along with MYOD, the transcription factors MY5, Myogenin, and MRF4 are responsible for the myogenic response in skeletal muscle (20).

Putatively, intrinsic sex differences in the transcriptional regulation of gene expression during fetal development may lead to dimorphisms in embryonic myogenesis and in utero muscle growth. Recent work in bovines has revealed novel sex dimorphisms in skeletal muscle development during intrauterine life, as indicated by a higher number of muscle cells and the upregulation of genes involved in myogenesis, adipogenesis, and fibrogenesis in muscles of male fetuses compared with female fetuses (5). Maternal factors have been investigated as an epigenetic factor during this intrauterine period. Although maternal overnutrition increases muscle protein content and muscle mRNA expression of myogenic markers (*CTNNB1*), adipogenic markers (*ZNF423*, *PPARG*), and fibrogenic markers (*FN1*), these variables are not influenced by fetal sex in bovines (5).

In contrast, maternal obesity differentially alters the skeletal muscle transcriptome in offspring of obese mice, with muscles of male fetuses displaying an enrichment of pathways pertaining mostly to metabolism, whereas muscles of female fetuses display an enrichment of pathways primarily in RNA synthesis and cellular transport (6). However, these transcriptomic responses to maternal obesity do not remain consistent throughout development as there is little overlap of differentially expressed genes between the fetal and 3-mo-old offspring of obese mums in either sex (6). Hence, the mechanisms dictating metabolic processes following exposure to maternal obesity are sexually dimorphic and evolve throughout skeletal muscle development. Taken together, not only do maternal factors play a role in differentially regulating gene expression between sexes during skeletal muscle development, but fetal sex is also an intrinsic determinant of prenatal muscle growth in mice.

Although studies examining sex differences during in utero muscle growth are admittedly scant, these current findings offer novel insights on muscle development for food scientists and other individuals in fields traditionally outside of the realm of developmental biology. Moreover, the discrepancy between bovine and murine models described here illustrates the challenges of translating findings from animal models to humans due to heterogeneity among species.

### Sexual Dimorphisms in Postnatal Skeletal Muscle Growth

Following childbirth, there are three broad but distinct phases that encompass postnatal growth: infancy, childhood, and puberty (21). The infancy (neonatal) stage of muscle development lasts approximately the first 6 mo of a child’s life, during which growth is a continuation of in utero development and is influenced primarily by nutrition rather than growth hormones (21). This timepoint at 6 mo also represents the average weaning age for infants and is equivalent to 28 days or 4 wk of age in mice (22). Regardless of delineation, the neonatal period of development is characterized by prodigious growth, with skeletal muscle being the organ that grows most rapidly in neonates. This rapid muscle growth is attributed to the frequent feeding that occurs during infancy, which activates the insulin signaling pathway (PI3K/Akt), leading to upregulation of mTOR signaling and the subsequent stimulation of muscle protein synthesis (23). Rapid satellite cell-mediated myonuclear accretion further sustains this substantial increase in skeletal muscle growth (23). Despite these integral events, the neonatal/early postnatal period is an often overlooked aspect of muscle development, and warrants in-depth investigations to prevent and treat pediatric muscle disorders.

Sex differences in muscle growth emerge at 4 wk in rodents, with neonatal male muscles displaying increased mass, myofiber cross-sectional area (CSA), and protein content than neonatal female muscles (7–9). Recent studies exploring novel strategies to treat pediatric neuromuscular contractures have serendipitously revealed putative mechanisms involved in this observed sex dimorphism in neonatal muscle growth. Neonatal denervation is known to disrupt postnatal skeletal muscle growth, and recent advances have implicated a disruption of proteostasis, or balance between protein synthesis and protein degradation, in this pathophysiological process (24). To restore proteostasis in denervated muscles, myostatin (MSTN), a member of the transforming growth factor-β (TGF-β) superfamily that functions as a potent negative regulator of muscle mass and mTOR-mediated protein synthesis (25), was pharmacologically targeted. Blockade of ligand binding of Mstn to its type 2 receptors with a soluble decoy receptor, activin receptor type 2B with an Fc tag (ACVR2B-FC) (26), enhances growth in CSA, length, volume, and mass in denervated muscles exclusively in female mice (8). Furthermore, although inhibition of Mstn signaling also improves growth parameters in the nondenervated muscles in both female and male mice, the effect is greater in female mice (8). Thus, sex differences in neonatal muscle growth may involve differential roles of MSTN between sexes. In contrast, chemical ablation of sympathetic neurons decreases CSA and volume only in noninjured muscles of male 4-wk old mice, suggesting a sex-specific requirement of sympathetic innervation for neonatal muscle growth (9). This sex-specificity is complemented by increased CSA and volume solely in noninjured male muscles of 4-wk old mice and rats upon β-adrenergic stimulation with clenbuterol, a β_2_-agonist sympathomimetic agent (9). These latter findings suggest that β_2_-adrenergic signaling mediates neonatal growth exclusively in male muscles. In all, the recent discoveries highlight sex-divergent roles of prominent signaling pathways in the development of neonatal skeletal muscles, offering unique molecular targets for both pediatric female and male patients with muscle diseases. Additional work is required to decipher potential contributions of other molecular signaling pathways, as well as to identify novel factors that mediate sex dimorphisms in muscle growth during the neonatal/early postnatal period.

Following the neonatal period, postnatal growth during childhood is mainly growth hormone dependent and driven by insulin signaling (21). In children, this switch from nutritional-dependent to hormonal-mediated growth occurs after 6 mo of age (21) and precedes the onset of puberty at around 11–12 yr (22). This intermediary stage of development coincides with a period of between 28 days/4 wk and 42 days/6 wk in mice (22). The last phase of postnatal growth encompasses puberty/adolescence in its entirety and is intricately mediated by the actions of sex steroids. The completion of growth plate closures marks the transition from adolescence to adulthood, with epiphyseal plates in the scapula fusing last at 17–23 yr in humans (27). This average age of 20 yr in humans coincides with sexual maturity at 10 wk in mice (22).

**Figure 1.**
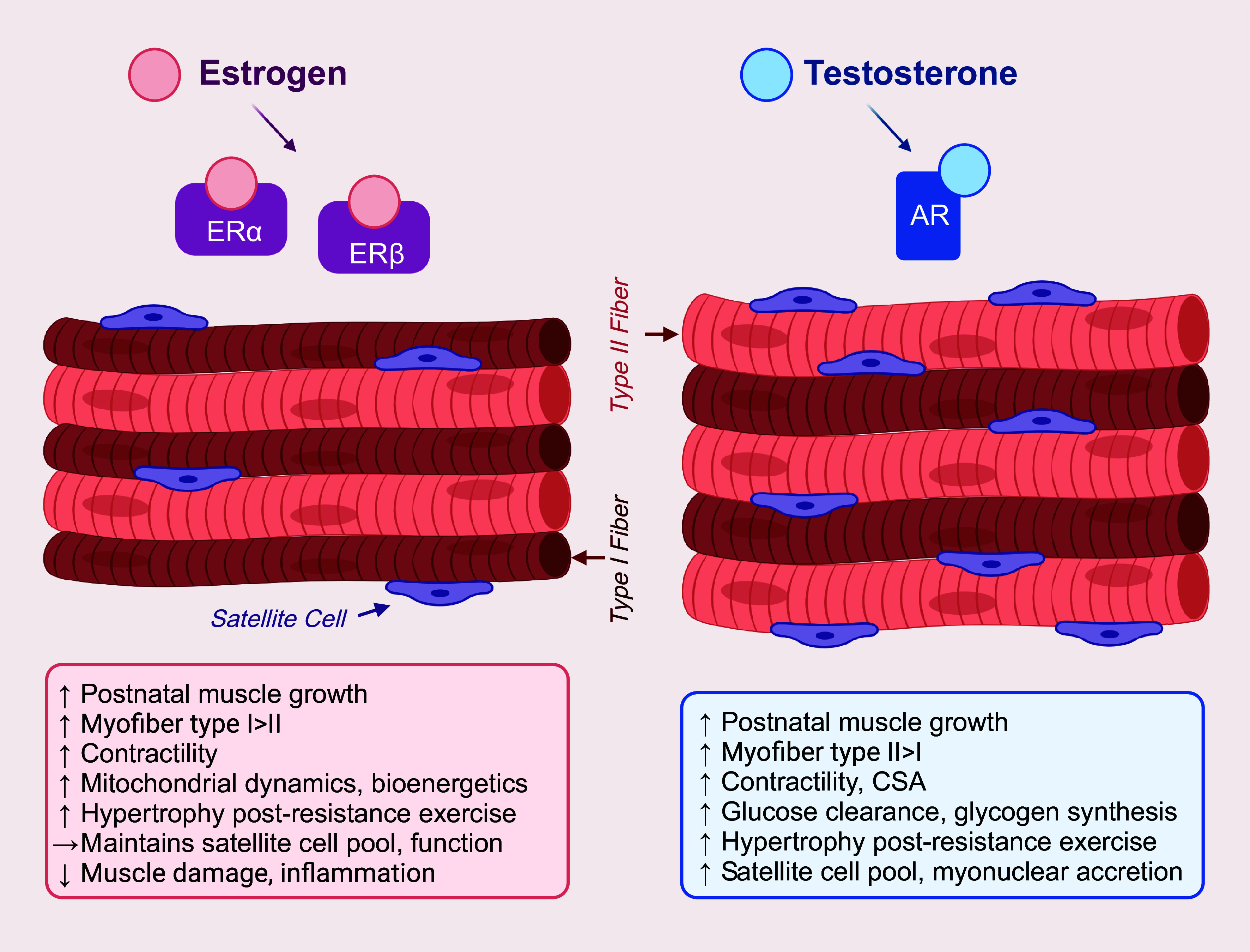
Sex hormone-mediated signaling complexes in skeletal muscle. Ligand binding of the primary sex steroids, estrogen and testosterone, to their respective nuclear estrogen receptors (ERα and ERβ) and nuclear androgen receptors (AR) elicits multiple downstream effects on muscle health and function. The estrogen-receptor signaling complex promotes postnatal muscle growth, facilitates muscle homeostasis by maintaining fiber-type distribution, mitochondria dynamics and bioenergetics, and the size of the satellite cell pool, regulates force generation and muscle contractility, governs the hypertrophic response to resistance exercise, and protects against muscle damage and inflammation. In comparison, testosterone-mediated androgenic signaling promotes postnatal muscle growth, facilitates muscle homeostasis by maintaining fiber-type distribution, upregulates glucose clearance and glycogen synthesis, augments satellite cell activity and myonuclear addition to the myofiber, enhances muscle mass and strength, and governs the hypertrophic response to resistance exercise. Note that while these two sex hormones regulate key physiological processes across both sexes, this schematic only depicts the effects of estrogen and testosterone signaling in female and male muscles, respectively. Created with BioRender.com.

During the pubertal phase of postnatal development, growth factors and sex hormones mediating muscle growth have long-term ramifications in muscle size and function at adulthood. In particular, estrogen is a steroid hormone that binds to α and β receptor subtypes (ERα, ERβ, respectively), whereas testosterone is a cholesterol-derived steroid hormone that binds to the androgen receptor (AR; NR3C4). Ligand binding of these sex steroids to their respective nuclear receptors leads to numerous downstream effects in skeletal muscle (Fig. 1). Gonadectomy in 3-wk-old mice impairs myofiber CSA in assorted muscles of female and male mice at 8 wk of age (7), suggesting a critical role for estrogen and testosterone in mediating muscle growth during the intermediate and early pubertal phases of postnatal development. However, the losses in myofiber size of both female and male mice are restored with dihydrotestosterone, but not estradiol (E2) therapy (7), indicating differential effects of sex steroids in postnatal muscle growth.

Although ovariectomy in juvenile 6-wk-old female mice does not decrease muscle growth throughout the rest of puberty, it attenuates myofiber CSA in hindlimb muscles after sexual maturity at 14 wk of age, and further attenuates grip strength at 30 wk of age (10). The temporal deficit in muscle growth is completely restored with administration of E2 in ovariectomized mice (10). These findings indicate a regulatory role of estrogen in facilitating female muscle growth and function during puberty and into adulthood. The importance of estrogen in postnatal muscle development is extended by more recent discoveries that myofiber ablation of *Erβ* at the onset of puberty (6 wk) decreases grip strength, and muscle weight and myofiber CSA of tibialis anterior muscles in female but not male mice at sexual maturity (10–12 wk) (11). These novel insights thus establish the estrogen-Erβ pathway as a sex-specific regulatory mechanism that governs late postnatal muscle growth in female mice (11). In male mice, orchiectomy-induced depletion of testosterone at 6–8 wk of age reduces muscle weight and myofiber CSA of assorted forelimbs, pelvic, and hindlimb muscles upon sexual maturity (∼10 wk) (12–14). These impairments in muscle growth are restored with testosterone supplementation, through modulation of pathways regulating glucose and nutrient uptake, as well as proteasomal and lysosomal activity (13, 14). These results illustrate the integral role of testosterone in mediating male muscle growth during adolescence. Sex differences in MSTN signaling also manifest at this pubertal stage, albeit in a manner that is both contradictory to and consistent with the neonatal period described earlier. Specifically, constitutive null mutation (*Mstn*^−/−^) promotes a more robust increase in triceps muscle weight of 2-mo-old male than female mice (15), which contrasts the aforementioned reports of greater increases in size and protein content of 4-wk-old female muscles after ACVR2B-Fc treatment (8). Conversely, when compared with respective *Mstn*^+/+^ controls at 4 mo of age, *Mstn* deletion increased myofiber CSA in soleus, plantaris, and gastrocnemius of *Mstn*^−/−^ female mice, and myofiber CSA only in soleus of *Mstn*^−/−^ male mice (16). Notwithstanding methodological differences between genetic and pharmacologic inhibition, these conflicting sex-specific findings at distinct stages of postnatal development posit a sex-dependent, muscle group-dependent, and temporally precise role for MSTN signaling throughout postnatal skeletal muscle growth.

## SKELETAL MUSCLE HOMEOSTASIS

The skeletal muscle system plays a central role in the maintenance of homeostasis within the body. Through the contraction of myofibers, skeletal muscle generates heat and regulates movements. Skeletal muscle relies upon energy to produce muscular contractions. As such, careful regulation of metabolic processes is critical to the maintenance of skeletal muscle homeostasis. However, progressive loss of skeletal muscle can result in dysregulation of homeostasis. In this section, we will highlight sex differences in the maintenance of muscle homeostasis, by exploring the influence of sex on muscle composition, muscle metabolism, and age-induced changes (Table 2).

**Table 2.** Summary of reported sex differences in the domain of skeletal muscle homeostasis

Muscle Domain	Species	Female	Male
*Muscle composition*			
• Fiber-type distribution			
Baseline (2, 28–35)	Human (2, 28–32), Mouse (33, 34), Rabbit (35)	↑ Type I fibers (human)	↑ Type II fibers (human)
		↑ Type IIA fibers in masseter (mouse)	↑ Type IIB fibers in masseter (mouse)
		↑ Type I fibers in masseter (rabbit)	↑ Type IIA fibers in masseter (rabbit)
Hypothyroidism (36)	Human	↑ Type II fibers in vastus lateralis vs. sex-matched controls	No observed phenotype vs. sex-matched controls
Ovariectomy (10, 37)	Mouse (10), Rat (37)	Shift toward type IIB fibers in tibialis anterior (mouse) and plantaris (rat) vs. sex-matched controls, restored with E2	N/A
Testosterone supplementation (38–40)	Human (Male) (38, 39), Rabbit (Male) (40)	Not studied	No change in vastus lateralis muscles vs. sex-matched controls (38) and vs. baseline levels (39) (human),
			Reverses metabolic syndrome-induced type II shift in vastus medialis vs. sex-matched controls (rabbit)
Myofiber deletion of *Ar* (41)	Mouse	No observed phenotype vs. sex-matched controls	Shift toward type I fibers in soleus vs. sex-matched controls
• Fiber size and function			
Baseline (28–31, 42–47)	Human	Slower myofiber phenotype facilitate fatigue resistance	↑ CSA across all fiber-types vs. female
			Faster myofiber phenotype facilitate force generation
Menopause (48)	Human	↓ Satellite cell numbers vs. baseline (perimenopausal) levels	N/A
Ovariectomy (10, 48, 49)	Mouse	↓ Satellite cell expansion, differentiation, renewal vs. sex-matched controls	N/A
SC deletion of *Erα* (48) or *Erβ* (11)	Mouse	↓ Satellite proliferation; ↑ apoptosis of satellite cells vs. sex-matched controls	Not studied (Erα), ↓ satellite proliferation (Erβ) vs. sex-matched controls
Myofiber deletion of *Erα* (50) or *Erβ* (11)	Mouse	↓ Muscle contractility vs. sex-matched controls	Not studied (Erα), no effect on muscle contractility (Erβ) vs. sex-matched controls
Testosterone supplementation (39, 51–59)	Human (Male) (39, 51–57), (Female) (58, 59)	↑ Lean mass, CSA, satellite cell, capillarization of type II fibers vs. sex-matched controls (58, 59)	↑ Satellite cell expansion, myonuclear accretion, myofiber CSA, muscle contractility vs. sex-matched controls (51, 52, 55–57) and vs. baseline levels (39, 53, 54, 57)
Orchiectomy (12)	Mouse	N/A	No effect on muscle weight, myofiber CSA, rates of protein synthesis in hindlimb muscles vs. sex-matched controls (adult and old mice)
• Fiber content			
Baseline (45, 60, 61)	Human (45), Mouse (60, 61)		↑ Satellite cell numbers in type II fibers of vastus lateralis (human) and myofibers of extensor digitorum longus (mouse) vs. female

Muscle Domain	Species	Female	Female and Male	Male
*Muscle metabolism*				
• Bioenergetics (62–67)	Human	↑ Reliance on β-oxidation		↑ Reliance on glycolysis
Substrates (63, 64, 68–71)	Human (63, 64, 68–70), Mouse (71)	↑ IMTG (63, 64);		
		↑ Lipid binding transport proteins (68–70);		
		↑ Mitochondria protein (71) vs. male		
Mitochondrial function (72)	Human	↓ ADP sensitivity; ↑ malonyl-CoA sensitivity vs. male	Mitochondrial proteins and electron transport chain complex	
Muscle transcriptome (7, 70)	Human (70), Mouse (7)	↑ Gene and protein expression of FAT/CD36 vs. male at baseline (70)		↑ Myofiber expression of *Pfkfb3* (7)
		↑ Myofiber expression of *Pdk4* (7)		
• Myofiber deletion of *Erα* (73)	Mouse (Female)	↓ Mitochondrial DNA replication and fission dynamics vs. sex-matched controls		Not studied
• Ovariectomy + E2 therapy (74)	Mouse	↓ Mitochondrial membrane microviscosity vs sex-matched controls to baseline proestrus levels		N/A
• Myofiber deletion of *Ar* (75)	Mouse	No effect on glucose metabolism vs. sex-matched controls		↓ Muscle glucose clearance, hexokinase activity, lipolysis vs. sex-matched controls
• Orchiectomy + Testosterone (76–78)	Mouse (76, 77), Rat (78)	N/A		↑ Muscle glycogen synthesis and content
*Muscle aging*				
• Sarcopenia (79–95)	Human	↑ Prevalence with earlier onset vs. male (85, 88, 89)	↓ Muscle mass and function (79–83)	Greater ↓ in absolute muscle mass vs. female (88, 93–95)
		↑ Rate of decline in muscle mass starting at 5th decade vs. male (86, 87)	Onset as early as 3rd decade (84, 85)	
		↑ Incidence of muscle injury and frailty vs. male (89–92)		
• Sex hormones (81–83, 85, 96–98)	Human	↑ Loss of estrogen starting at 5th decade (85, 96–98)	↓ Endogenous levels (81–83)	
• Proteostasis				
Baseline (99–101)	Human	Basal muscle protein FSR in nonobese aged female:		Basal muscle protein FSR in nonobese aged male:
		↓ vs. nonobese young female (99)		↓ vs. nonobese young male (99)
		↑ vs. nonobese young female, +0.014%/h (100)		No change vs. nonobese young male (100)
		↑ vs. nonobese aged male (99), +0.017%/h (100)		
		↑ Basal muscle protein FSR in obese aged female vs. obese aged male (101)		
Nutrition (100, 101)	Human	No change in muscle protein FSR with glucose, insulin, amino acid infusion in nonobese aged female vs. basal levels (100)		↑ Muscle protein FSR with glucose, insulin, amino acid infusion in nonobese aged male vs. basal levels (100)
		No change in postmeal muscle protein FSR in obese aged female vs. basal levels (101)		↑ Postmeal muscle protein FSR in obese aged male vs. basal levels (101)
Exercise (102, 103)	Human	↑ Muscle protein FSR vs. baseline levels after a 3-mo exercise program (102)	Shift toward type IIA fibers after 26-wk resistance training program (103)	Greater ↑ in muscle protein FSR vs. baseline levels after a 3-mo exercise program (102)
		Modest ↑ in myofiber CSA and relative strength vs. baseline levels after 26-wk resistance training program (103)	Shift toward type IIA fibers after 26-wk resistance training program (103) ↑ myofiber CSA and relative strength vs. baseline levels and vs. female after 26-wk resistance training (103)	↑ myofiber CSA and relative strength vs. baseline levels and vs. female after 26-wk resistance training (103)
• Inflammation (83, 95, 104–106)	Human		↑ TNFα and IL-6 levels with loss of estrogen and testosterone	
• Autophagy-lysosome system (71, 107)	Mouse	Greater ↓ in mitochondrial capacity; greater ↑ in activation of autophagy-lysosome pathway vs. aged male (71)	↑ Autophagy, mitochondrial density and capacity with resistance exercise (107), no change with aerobic exercise (71)	↓ Mitochondrial content vs. young male and aged female (71)
• Satellite cells (108, 109)	Human	Maintain expansion capacity and oxidative phosphorylation capacity vs. young female (109)	↓ Number and function/regenerative capacity	↓ Expansion capacity, ↑ secondary apoptosis,
				↓ Oxidative phosphorylation vs. young male (109)

AR, androgen receptor; CSA, cross-sectional area; E2, estradiol; ERα, estrogen receptor alpha; ERβ, estrogen receptor beta; FSR, fractional synthesis rate; IMTG, intramuscular triglyceride; Pdk4, pyruvate dehydrogenase kinase 4; Pfkfb3, phosphofructokinase-2.

### Sexual Dimorphisms in Skeletal Muscle Composition

Skeletal muscle fibers consist of the following four primary classifications: type I, type II-A, type II-X, and type II-B fibers, which are determined by expression of myosin heavy chain (*MYH*) isoforms (110). Each fiber type also varies based on its myoglobin content, rate of contraction, metabolic processes used to generate ATP, and fatigue onset (111). Briefly, type I fibers express *MYH7*, contain abundant myoglobin and mitochondria, and predominantly utilize aerobic metabolism as an energy source. As a result, type I fibers generate high levels of ATP, produce slow contractions, and are highly resistant to fatigue. In contrast, type II fibers contain less myoglobin and fewer mitochondria, but are replete in glycogen stores, and utilizes anaerobic metabolism (glycolysis) more efficiently to generate ATP for shorter and faster contractions (112). However, these fibers fatigue easily during intense contractile activity presumably due to excess lactic acid accumulation stemming from the increased reliance on anaerobic metabolism, which leads to an attenuation of Ca^2+^ release from the sarcoplasmic reticulum and decreased Ca^2+^ sensitivity, thereby impairing the cycling of actin-myosin cross-bridges (113, 114). Type II fibers can be further delineated into the following subtypes, in ascending order of contraction velocity and descending order of fatigue resistance: Type IIA (*MYH2*), Type IIX (*MYH1*), Type IIB (*MYH4*). It is paramount to consider that the different fiber types and their metabolic properties are not conserved across mammalian species. Due to functional demands, limb muscles of smaller mammals such as rodents and rabbits exhibit high proportions of Type IIX and IIB fibers. In contrast, these fiber types are rarely present in the limb muscles of larger mammals (including healthy adult humans), which are composed primarily of type I and IIA fibers (2, 112, 115). Moreover, there is species diversity in the metabolic properties and contractile kinetics of fiber types, presumably due to pronounced differences in body mass. Compared with rodents, the increase in body mass of larger mammals, including humans and bovines, is associated with a lower abundance of myofiber oxidative enzymes as determined by mitochondrial volume density (112, 116, 117), as well as reduced substrate oxidation as determined by mitochondrial citrate synthase activity (112, 118). Furthermore, myofibers of rodents and rabbits display a higher maximal velocity of shortening (112, 119, 120) and a more rapid contraction time (112) than their human and bovine counterparts. This heterogeneity in fiber-type distribution, metabolic properties, and contractile kinetics are further confounded by observations of sex dimorphisms unique to individual species.

At the myocellular level, male humans have a greater proportion of type II fibers compared with female humans, regardless of physical training level (28, 29). Correspondingly, female humans have a higher distribution of type I *MYH* isoforms, with type I fibers also constituting a higher percentage of the biopsy area sampled (2, 30). At the transcriptional level, vastus lateralis muscles of female humans express 35% more *MYH7* RNA but 30% less *MYH2* RNA than corresponding male muscles (31). In the same muscle group, type I fibers account for 44% of the total biopsy area in female humans, compared with 36% in male humans (32). It is presently unclear if this dimorphism observed in humans is conserved across muscle groups (30). Furthermore, although sex differences in fiber-type distribution have previously been reported in murine and rabbit models, these findings were limited to the masseter muscle (33–35). Although there is a need to reconcile the differences across species and muscle groups, the underlying mechanisms for these reported sex differences within individual species remain unclear and need to be further elucidated as well.

It is postulated that the sex differences in fiber-type proportion is due, in part, to levels of sex steroids and thyroid hormone circulating in the bloodstream (2, 42), although concrete evidence of their roles in humans is currently limited. Clinically, the proportion of type II fibers may potentially be higher in the vastus lateralis of female humans with hypothyroidism than their male counterparts (36). In contrast, the evidence for hormonal regulation of sex differences in fiber-type distribution may be better established in animal models. In particular, estrogen has been shown to maintain fiber-type composition in muscles with predominantly type II fibers, as tibialis anterior of estrogen-depleted (ovariectomized) mice displayed a lower proportion of type IIA fibers concomitant with a shift toward a type IIB distribution (10). Similarly, in plantaris muscles of ovariectomized rats, the reduction in proportion of type IIX fibers (5.7%) was accompanied by a mild increase in type IIB fiber distribution (3.3%) (37). Treatment with E2 completely restored fiber-type proportions in mice and restored type IIX expression in rats (10, 37). Comparatively, the role of testosterone in fiber-type distribution is less clear. Although testosterone supplementation does not alter fiber-type proportion in vastus lateralis muscles of older male humans (38, 39), it reverses the shift toward a glycolytic type II phenotype induced by high-fat diet in vastus medialis muscles of male rabbits (40), indicating a potentially species-protective effect of testosterone in type I fibers against metabolic syndrome. In rodents, the maintenance of type II fibers in male muscles may instead be dependent on Ar function, as targeted ablation of *Ar* in myofibers induces a shift toward type I fibers and increases expression of slow-twitch fiber genes in soleus muscles of male mice (41). Future work is thus required to translate these findings in humans, as well as elucidate the roles of estrogen/type II fiber-type maintenance and testosterone/androgen-AR signaling in muscle composition.

In addition to fiber-type distribution, myofiber size is also influenced by sex. Previous studies have shown that myofiber CSA is greater in male humans than in female humans across all fiber types (28–31, 42). Consequently, not only do female human muscles comprise a greater proportion of slow oxidative muscle fibers, but their fast glycolytic fibers are smaller as well (43–47). Although the overall faster myofiber phenotype in male humans may facilitate rapid movements and force generation, the slower myofiber phenotype in female humans may provide fatigue resistance (62, 121). These discrepancies in size and function may be potentially attributed to sex differences in satellite cell content and myonuclear composition among the fiber types. Although satellite cells maintain muscle homeostasis by mediating repair and remodeling processes, current understanding of precise mechanisms underlying these processes is limited by heterogeneity of the satellite cell compartment (122). Broadly, satellite cells are activated during skeletal muscle damage and give rise to myogenic progenitors (18), which subsequently proliferate, differentiate, and migrate to the site of injury to fuse into the existing damaged myofiber and donate their nuclei to help with the repair response, or fuse with one another to form nascent myofibers with functional contractile apparatus (123). Earlier studies reported a lack of sex differences in satellite cell abundance in the tibialis anterior and vastus lateralis of both physically active and untrained adult humans (44, 46, 47, 124). When further delineated by fiber type, more recent findings indicate that type II fibers in vastus lateralis of adult untrained male humans may contain more satellite cells than female counterparts (45). These baseline sex discrepancies in fast fiber types of humans are corroborated by murine models that reported a greater number of satellite cells in the extensor digitorum longus (which comprise predominantly type II fibers) of adult male mice compared with their female counterparts (60, 61). Additional studies are needed to validate this intriguing sex difference in fiber composition.

The impact of testosterone on muscle size in males is well-documented across different age groups and training paradigms. In male human myofibers, testosterone augments satellite cell expansion, myonuclear accretion, and CSA (39, 51–55). The cellular mechanism for these androgenic actions putatively involves an upregulation of follistatin (an inhibitor of MSTN and other TGF-β family members) and concomitant inhibition of TGF-β signaling in mouse satellite cells (125). The anabolic effects of testosterone also extend to muscle contractile function, as testosterone supplementation in older male humans increases isometric knee extension peak torque (56), and also enhances peak power of both type I and type II fibers in vastus lateralis muscles (57). In addition, although characterization of the ergogenic effects of anabolic hormones have been lacking in females (126), moderate doses of testosterone in young female humans has recently been shown to improve aerobic performance and lean mass (58), and increase myofiber CSA, satellite cell content, and capillary content of type II fibers (59). Despite these therapeutic benefits, testosterone may not be required to maintain muscle mass past sexual maturity, as orchiectomy does not alter the weight, myofiber CSA, and rates of protein synthesis in hindlimb muscles of adult and old male mice (12).

In female myofibers, estrogen plays a critical role in preserving size and function by maintaining the satellite cell pool, as its deficiency impairs numerous myogenic processes. Specifically, satellite cells isolated from muscles of ovariectomized female mice and/or postmenopausal female humans display a decline in numbers (10, 48), a defect in S-phase and G2/M-phase entry of the cell cycle (49), a reduced proportion of differentiating and self-renewing cells in vitro (10), and an impaired ability to differentiate into myofibers in vivo (48). Several findings have recently emerged to elucidate the mechanisms by which autonomous estrogen signaling preserves the satellite cell compartment. In satellite cells of female mice, ablation of *Erα* and *Erβ* independently induces apoptosis, with *Erα* deletion altering the expression of genes promoting cell death and survival (48), and *Erβ* deletion altering the expression of genes involved in cell-cycle progression (11). These findings indicate a protective role of estrogen-receptor signaling against satellite cell loss. Myofibers and whole muscles of ovariectomized female mice, *Erα*-deficient female mice, and *Erβ*-deficient mice all demonstrate reduced force generation and contractility, further underscoring the importance of this signaling complex for skeletal muscle function (10, 11, 50). Overall, these recent findings suggest that, even at the myocellular level, sex-based differences in skeletal muscle composition exist.

### Sexual Dimorphisms in Skeletal Muscle Metabolism

Skeletal muscle is a highly metabolic organ sustained by the precise regulation of a series of ATP-producing biochemical reactions. These bioenergetic pathways allow for skeletal muscle to perform its role in locomotion as well as maintain systemic health and longevity. Recent evidence has suggested that skeletal muscle metabolism is regulated in a sex-dependent manner. A closer look into these sexual dimorphisms is necessary for a more complete understanding of the influence of sex on muscle bioenergetics under various conditions.

Although female muscles display properties that are conducive to aerobic metabolism, they may not involve mitochondrial substrate transport, as paradoxically, resting muscles of female humans are less sensitive to mitochondrial ADP, but more sensitive to malonyl-CoA (an inhibitor of mitochondrial fatty acid uptake) compared with male humans (72). Despite this, the composition of myocellular substrates differ between the sexes, as female humans store higher levels of intramuscular triglycerides (IMTG) at rest (63, 64). The underlying mechanism(s) for this sex discrepancy is currently unclear. Fiber-type composition may be a contributing factor, as IMTG content has been reported to be threefold higher in type I than type II fibers of healthy male humans (65, 66). Although there is an increased proportion of type I fibers in female humans, it is not clear whether female muscles exhibit this pattern of fiber-type disparity in lipid content. Another potential contributing factor is a higher expression of lipid binding transport proteins, such as FAT/CD36, FATP1, and FABPpm, in female than male humans (68–70). In particular, plasma-membrane associated FAT/CD36 has been postulated to be highly correlated with IMTG storage in human muscle (127). Although gene and protein expression of FAT/CD36 has been reported to be higher in female muscles (70), the amount of sarcolemma-bound FAT/CD36 in female humans has not been investigated conclusively. Regardless of mechanisms, there is a greater reliance on IMTG as an energy source during exercise in females than males (62–66). Thus, the sex discrepancies in myocellular composition may underlie why female skeletal muscle preferentially utilizes mitochondrial fatty acid β-oxidation whereas male skeletal muscle relies upon glycolysis (62, 67).

To investigate this phenomenon further, a recent loss-of-function study of cultured type II-B muscle fibers revealed that *Pfkfb3* (phosphofructokinase-2) and *Pdk4* (pyruvate dehydrogenase kinase 4) genes serve as switches between the two sexually dimorphic bioenergetic pathways (7). The glycolytic pathway utilizes PFKFB3 as a mediator for the conversion of fructose-6-phosphate into fructose-2,6-bisphosphate. Male rodent muscle fibers have a greater expression of *Pfkfb3*, providing a genetic basis for the increased reliance on glycolysis in male muscles. Conversely, PDK4 is a regulator of fatty acid β-oxidation, and E2-induced expression of the *Pdk4* gene enhances fatty acid-dependent mitochondrial oxygen consumption in female rodent muscle fibers (7) (Fig. 1). However, the loss of *Pdk4* negates E2 enhancement in female rodent muscles. Thus, E2-induced *Pdk4* gene expression may potentially underlie the reliance on fatty acid β-oxidation in female muscles (7). Given that E2 levels fluctuate throughout a female’s lifespan, especially during the reproductive cycle, these findings provide insight into the influence of hormones on female skeletal muscle metabolism.

Unsurprisingly, the sex hormones also play a pivotal role in regulating substrate utilization. The effects of androgens on glucose homeostasis in skeletal muscle are well-established, as castration in male rodents depletes muscle glycogen levels, whereas testosterone therapy enhances muscle glycogen synthesis and content (76–78). Testosterone also mediates the expression of key proteins and enzymes involved in muscle glucose uptake (GLUT4) and glycolysis (phosphofructokinase and hexokinase) in myoblasts of rats (128). These anaerobic functions are coordinated through the androgen/AR axis, as myofiber deletion of *Ar* impairs glucose clearance and hexokinase activity in muscles of male mice, whereas dihydrotestosterone enhances glycolysis in C2C12 cells (75). Furthermore, androgen signaling also regulates muscle oxidative metabolism, as evident by decreased lipolysis in *Ar*-deficient myofibers (75) and increased mitochondrial turnover in skeletal muscles of castrated male mice (129). In contrast, estrogen controls metabolic homeostasis in skeletal muscle by protecting mitochondrial function (130). *Erα* ablation in myofibers of female mice diminishes muscle oxidative metabolism by impairing mitochondria DNA replication and fission dynamics (73), whereas E2 localizes to mitochondria membrane independently of Erα, improving bioenergetic function and lowering membrane microviscosity in muscles of female mice (74).

### Sexual Dimorphisms in Aging Skeletal Muscle

Protein turnover reflects the balance between continuous protein degradation and synthesis, a process essential for the maintenance of muscle proteostasis. When protein catabolism exceeds anabolism, skeletal muscle mass is in disequilibrium, ultimately leading to muscle wasting. Although catabolic states often occur under diseased conditions, muscle homeostasis can also be altered in the absence of disease. The loss of muscle mass and function with aging are known as sarcopenia and dynapenia, respectively (79). Since skeletal muscle is critical to the motor function and metabolic regulation of the body, sarcopenia is associated with a higher incidence of falls, hospitalizations, and comorbidities (80). With advancing age, endogenous levels of estrogen and testosterone decline in postmenopausal female humans and elderly male humans, respectively, and are associated with a host of deleterious outcomes, including muscle loss and dysfunction (81–83). In particular, the decline in estrogen during menopause profoundly impacts sarcopenia in a sex-specific manner. Although declines in muscle mass occurs at a rate of 0.4–0.8 kg per decade starting at the third decade of age (84, 85), female humans experience an exacerbated decline of 0.6% per year of muscle mass starting at the fifth decade (86, 87), coinciding with the loss of estrogen (85, 96–98). Perhaps unsurprisingly, sarcopenia is more prevalent and occurs earlier in female than male humans (85, 88, 89). The accelerated loss of estrogen with menopause in female humans is further associated with a greater incidence of skeletal muscle injury and clinical frailties (89–92). In contrast, contemporary literature generally agrees that loss of muscle mass is greater in aged male humans, even after accounting for absolute differences in initial muscle mass (88, 93, 94), as male humans ≥75 yr old lose muscle mass at a rate of 0.80–0.98% per year compared with 0.64–0.70% per year in female counterparts (95). The mechanistic role(s) of sex hormones in mediating these discrepancies remains to be delineated (104).

The loss of muscle mass with aging is attributed primarily to an imbalance between muscle protein synthesis and muscle protein breakdown (85, 131). Recent advances have augmented our understanding of how aging alters muscle protein metabolism. Earlier studies using stable-isotope tracers in human subjects found reductions in myofibrillar protein synthesis with aging (132), and decreases in whole body muscle protein synthesis as early as ∼50 yr of age (133). This latter reduction in basal postabsorptive protein turnover in middle-aged adults is accompanied by decreased synthesis of mitochondrial and contractile proteins, but not sarcoplasmic proteins (133, 134). As several later studies reported mixed success in replicating these prior results (135–138), the notion of decreased muscle protein metabolism in the basal postabsorptive state with aging generally receives less support among contemporary views (139, 140). Despite this ambiguity, sex differences have been reported in basal muscle protein synthesis during aging. Among nonobese adults at baseline, older female humans are observed to have a higher rate of muscle protein synthesis than age-matched male humans, although comparisons with sex-matched young humans are mixed (99, 100). In addition, older, obese female humans also display a greater basal postabsorptive muscle protein synthesis compared with their male counterparts (101). It should be noted that in these instances of reported sex dimorphisms, protein metabolism was assessed by protein fractional synthesis rate (FSR), which calculates the rate of labeled amine acid precursors, thereby offering insights on metabolic adaptations to processes regulated by protein levels (141). Beyond sex dimorphisms, the disparate comparisons with young humans among nonobese adults (99, 100) further obscure whether muscle protein synthesis is lost with aging. Instead, current literature generally supports a diminished response of the aged muscle to protein ingestion or anabolic stimuli, a phenomenon coined “anabolic resistance” (142, 143). Thus, contemporary approaches have focused on modifying both nutritional intake and physical activity levels to mitigate this blunted response in older adults (139, 140, 144).

The benefits of exercise and nutrition on skeletal muscle function and overall health in elderly individuals are well-supported (79, 85, 139, 140, 144, 145). Not only are exercise and nutritional intake considered the primary modalities for establishing a net positive muscle protein balance, but they may further assist in overcoming aging-associated anabolic resistance (143). However, the degree of benefit that exercise and nutrition confer to aging muscle may be dependent on a person’s sex, as assessed by FSR in older humans (100–102). In nonobese humans, infusion of glucose, insulin, and amino acids increases muscle protein FSR past baseline levels in older male but not in female humans (100). In addition, following a meal consisting of small amounts of protein (as determined by fat-free mass), muscle protein FSR is elevated in older, obese male but unchanged in older, obese female humans (101). It should be noted that female subjects ingested smaller amounts of protein (∼10 g) compared with their male counterparts (∼13 g). Furthermore, after 3 mo of exercise training, older, obese male humans experience a doubling in muscle protein FSR compared with a 40% increase in their female counterparts (102). Besides muscle protein levels, older male humans also exhibit greater myofiber hypertrophy and relative strength gains after 26 wk of resistance training (103). Given that older female humans do not experience the same stimulatory effects of exercise or feeding as older male humans, these collective results may suggest that the inhibitory effects of anabolic resistance are more pronounced in older female humans. This blunted anabolic response may be associated with the menopausal decline in sex hormonal status (146, 147). Specifically, estrogen replacement therapy is associated with elevated levels of myofibrillar protein FSR and higher expression of myogenic genes in postmenopausal women after exercise (148, 149), indicating that estrogen deficiency may attenuate muscle sensitivity to anabolic signals during aging. Taken together, these findings posit that age may differentially regulate at least one common pathway in male and female muscles. Despite these reports of sex dimorphisms with aging, other studies have found no differences in muscle protein synthesis during basal postabsorptive conditions (150–153), the acute response to nutritional stimuli (151, 152), or following resistance exercise (152, 153). Although some of these discrepancies may be attributed to methodological differences in protein measurement and subject selection (154), future studies are warranted to foster a stronger consensus in this field.

In comparison, methodological constraints coupled with a reduced level of emphasis currently limit our overall understanding of muscle protein breakdown during aging (139). Despite this, chronic inflammation that is systemic and low-grade has been identified as a potential factor of muscle protein breakdown in aged humans (93, 95, 104, 155, 156). Specifically, the elevated levels of proinflammatory cytokines TNFα and IL-6, among others, in aged muscles may serve as putative catabolic signals that upregulate the ubiquitin-proteasome system, induce fat accumulation and muscle insulin resistance, and inhibit muscle protein synthesis (82, 95, 104, 105, 157). The loss of E2 and testosterone in postmenopausal female humans and aged male humans, respectively, are associated with increased levels of both TNFα and IL-6 (83, 95, 104–106), suggesting a protective role of sex hormones against the catabolic effects of these cytokines.

In addition to inflammation, recent findings from murine models may offer insights into the interaction between sex and aging in protein degradation. Specifically, skeletal muscles of adult male mice display fewer mitochondrial and lysosomal proteins, and less autophagosomal activity compared with female mice (71). As aging progresses, male mice exhibit a reduction in mitochondrial content whereas female mice exhibit relatively unchanged levels of mitochondria, although recent findings reported diminished mitochondrial respiration concomitant with greater autophagosomal clearance in aged female muscles (71). Although these insights on putative sex differences are informative, substantial work is required to more clearly delineate the impact of aging on mitochondrial respiratory capacity, a controversial topic at present. As exercise has been shown to initiate autophagosomal turnover (158, 159), it could serve as a potential modality to prevent sarcopenia by stimulating mitochondrial biogenesis and autophagy in aged muscles (107). However, the mode of exercise may determine the overall level of efficacy, and mask potential sex-specific responses. Although voluntary long-term resistance wheel exercise increases autophagy along with mitochondrial density and capacity in both aged male and female mice (107), acute treadmill running to exhaustion selectively stimulates autophagosomal breakdown in adult male mice, but not in adult female mice or in aged mice of either sex (71). These contradictory findings from a small number of murine studies may reflect methodological limitations when interpreting sex differences during aging, which include (but are not limited to) a paucity of aged ovariectomized mice (104). Therefore, there is a need for future work to clearly delineate the roles of sex in autophagy and other catabolic processes during aging, and in different species.

As an individual ages, the likelihood of a skeletal muscle injury increases. However, the capacity of human muscle progenitor cells to expand and maximize the number of cells available for muscle regeneration diminishes with age (108). Interestingly, muscle progenitor cells from older male human donors experience a reduction in expansion capacity and increased secondary apoptosis early in expansion compared with younger male donors, whereas female muscle progenitor cell expansion capacity is not affected by age (109). This sex difference in age-induced reduction of expansion capacity has been attributed to changes in metabolism. Although oxidative phosphorylation is impaired in muscle progenitor cells of older male donors, young and older female muscle progenitor cells maintain similar levels of oxidative phosphorylation and glycolysis markers (109). These results suggest that sex-dependent differences in substrate utilization potentially occur with age, and these metabolic alterations may underlie the sex-specific changes in expansion capacity of muscle stem/satellite cells. Further investigations are needed to validate the findings from this study.

## SKELETAL MUSCLE ADAPTIVE RESPONSES

A hallmark of skeletal muscle is its plasticity in response to external stimuli. Its robust capacity for adaptation and remodeling is important for muscle health and function, as well as the prevention of acute and chronic conditions. Exercise/physical activity, injuries, and disuse/inactivity all stimulate physiological skeletal muscle remodeling, leading to distinct morphologic and functional outcomes. In this section, we will discuss the role of sex in the muscle adaptive responses to each of these physiologic stimuli (Table 3).

**Table 3.** Summary of reported sex differences in the domains of skeletal muscle adaptive responses

Muscle Domain	Species	Female	Female and Male	Male
*Response to exercise*				
•Resistance exercise (160, 161)	Human	Higher capacity for upper-body strength gains?	↑ Relative gains in muscle hypertrophy and strength	↑ Absolute gains in muscle hypertrophy and strength
Muscle transcriptome (162)	Human	↑ Expression of atrophic signaling genes 24-h postexercise		Prolonged enrichment of differentially expressed genes, ↓ expression of inhibitors of hypertrophic signaling
Menopause (163, 164) + hormone therapy (165, 166)	Human	Greater ↑ in satellite cells and myofiber damage in postmenopausal female vs. young female and aged male. ↑ Body composition, muscle size, and contractile function in postmenopausal females on HRT or E2 therapy		N/A
Fiber composition/content (28, 43, 45)	Human		↑ Satellite cell content and CSA in Type II fibers	↑ Type I CSA of vastus lateralis vastus lateralis vs female (across different training modes and intensities) ↑ Myonuclear domains of Type I in vastus lateralis vs. female (resistance training to concentric failure)
•Eccentric exercise (167–169)	Human	↓ Muscle damage, inflammation with E2 therapy postmenopause vs. sex-matched control		↑ Inflammatory response and cytokine activity (167–169); ↑ Satellite cell activation (168) vs. female
*Response to muscle injury*				
•Cardiotoxin/Barium chloride-induced injury (170)	Mouse	↑ Removal of necrotic fibers; Greater ↑ in intramuscular fat deposition vs. male		↑ Intramuscular fat deposition vs. baseline levels
Gonadectomy (170)	Mouse	↓ Intramuscular fat deposition vs. nonovariectomized female		↑ Intramuscular fat deposition vs. non-castrated male
Satellite cell deletion of *Erβ* (11)	Mouse	Greater ↓ in muscle weight, ↓ myofiber CSA, ↑ intramuscular collagen deposition vs. sex-matched controls		↓ Muscle weight vs. sex-matched controls
*Response to disuse atrophy*				
•Short-term knee immobilization (171)	Human	Greater ↓ in isometric and concentric strength from baseline levels vs. male	↓ Muscle mass and CSA	↓ Isometric and concentric strength
•Hindlimb suspension/unloading (172, 173)	Mouse (173), Rat (172)	↓ Hindlimb grip strength vs. sex-matched control (Rat) ↓ Muscle mass and CSA of type IIB fibers in tibialis anterior; ↑ induction of catabolic factors and anabolic inhibitors vs. baseline (Mouse)	↓ CSA of type IIA/IIXD fibers in tibialis anterior vas baseline (Mouse)	↓ Hindlimb grip strength vs. baseline, vs. sex-matched control, and vs. female (Rat)
Ovariectomy (174, 175)	Rat	↓ Muscle mass recovery after reloading vs. sex-matched intact controls. E2 therapy restores myofiber regeneration and contractile function and to intact levels.		N/A
•Tenotomy (176)	Mouse	↓ Myofiber number vs. male	↓ Muscle mass	Greater ↓ in muscle mass, ↓ myofiber CSA vs. female ↑ intramuscular accumulation of large autophagic vesicles

CSA, cross-sectional area; E2, estradiol; HRT, hormone replacement therapy.

### Sexual Dimorphisms in Skeletal Muscle Adaptative Responses to Exercise

In contrast to age-associated protein catabolism, exercise alters muscle homeostasis by augmenting protein anabolism. Indeed, protein synthesis is essential for the repair and remodeling of muscles after exercise. Unfortunately, since female humans were often underrepresented in exercise training studies (177–179), much of our prior knowledge of the muscle adaptive response to exercise is derived from studies that only included male subjects or mixed cohorts without proper delineation between sexes (62, 180, 181). In this subsection, we will highlight select recent findings of sex dimorphisms in response primarily to resistance training and eccentric exercise.

When assessing exercise training adaptations between sexes, it is important to consider that the adaptive response is dependent on the initial training status of the subjects, as well as their basal levels of muscle strength and mass. In particular, the matching of subjects across sex is challenging based on general sex differences in initial muscle size and function. Therefore, caution must be exercised to discern absolute from relative changes when interpreting findings to clearly elucidate potential sex differences in the training response. However, emerging research has revealed several key differences between the sexes in the muscle adaptive response to exercise.

At the transcriptional level, the time course of gene regulation in skeletal muscle in response to acute resistance exercise potentially differs between the sexes. Following strenuous unilateral arm resistance training, male human subjects experience a prolonged alteration in their muscle transcriptomes, with Gene Ontology (GO) terms and Kyoto Encyclopedia of Genes and Genomes (KEGG) pathways significantly enriched with differentially expressed genes at 24-h postexercise (162). In contrast, the majority of significantly enriched GO terms and KEGG pathways were observed only at 4-h postexercise in the muscles of female human subjects, indicating a swift restoration to baseline. Moreover, negative regulators of mTOR signaling (a hypertrophic pathway), including *REDD1*, *REDD2*, and *PRAS40* were downregulated solely in male muscles, whereas genes related to SMAD binding, TGF-β signaling, and Notch signaling (pathways that induce atrophy or inhibit myogenesis) were upregulated primarily in female muscles, at 24-h postresistance exercise (162). Although these novel findings may provide some mechanistic insights to sex differences in muscle transcriptional regulation, additional studies are needed to validate and expand upon them. Moreover, it is imperative to elucidate the underlying mechanism(s) behind conflicting reports on the degree of muscle hypertrophy between male and female humans after resistance training (160, 161). Although absolute gains in muscle mass and strength are greater in male humans, the relative gains are similar between sexes (160), and relative strength changes in the upper body may even favor female humans (161).

At the hormonal level, the role of circulating hormones in mediating exercise-induced hypertrophy in male humans is presently unclear (182, 183). Specifically, it is unknown whether the hypertrophic response to resistance exercise in male muscles is mechanistically related to acute elevations in testosterone and other anabolic hormones (182, 184), or influenced by intramuscular androgen receptor content in lieu of circulating hormones (183, 185). In female humans, a 9-wk knee extension protocol facilitated greater improvements in quadriceps strength in premenopausal subjects, but led to a greater increase in the number of satellite cells, along with an increased presence of myofibrillar disruption in postmenopausal subjects (163, 164). Despite the potential for increased muscle damage, resistance exercise confers beneficial muscle adaptations to postmenopausal female humans. Hormone replacement therapy (HRT) in postmenopausal female subjects further enhances the muscle response to 1 year of weight-lifting exercises by decreasing fat tissue (165). Furthermore, estrogen therapy is able to further improve quadriceps CSA, handgrip strength, and lean body mass in postmenopausal female humans following 12 wk of lower-body resistance training (166). These select findings highlight the importance of circulating hormones in mediating resistance exercise-induced adaptations in female muscles.

At the cellular level, several prior studies have observed a more robust increase in myofiber hypertrophy of young adult male than female humans after resistance training, concomitant with a sex-specific increase in satellite cell expansion and/or myonuclear content (44, 46, 47). However, contemporary studies that delineate between fiber types may establish additional insights on the role of sex in training-induced myofiber adaptations. Following resistance training to fatigue or concentric failure in adult humans, type II fibers of vastus lateralis (which displays a mixed fiber-type composition) have recently been reported to experience similar increases in satellite cell content and CSA, irrespective of sex (43, 45). Conversely, resistance training may induce a sex-specific increase in type I CSA of vastus lateralis of adult male humans, independent of satellite cell expansion (43, 45). This observed sex discrepancy in type I fibers with heavy resistance training may potentially extend to other modes of exercise and/or training intensity. Across different modes of moderate-intensity exercise, including resistance training, endurance training, or a combination of both, the type I fibers in vastus lateralis of adult male humans display larger CSA compared with female counterparts, despite similar levels of satellite cell content (28). It should be noted that this sex discrepancy in the hypertrophic response of type I fibers to exercise training is observed mainly in younger adult humans (18–40 yr) (28, 43, 45). Middle-aged (40–64 yr), elderly (60–73 yr), and very old (83–94 yr) adult humans do not experience hypertrophy of type I fibers with resistance exercise, independent of sex (186–188).

Among younger adults, recent findings also disagree on the adaptive response in type II fibers between sexes, as the vastus lateralis of moderately trained adult female humans exhibit a diminished satellite cell content in the type II fibers compared with moderately trained adult male humans, which coincides with smaller type II fibers in female than male muscles (28). Whether the sex discrepancies in satellite cell abundance and CSA of type II fibers are intrinsic in this cohort or directly induced by moderate training across different modes of exercise is inconclusive, as baseline measurements were not reported in this study. Regardless, these results contrast contemporaneous reports of sex-independent increases in satellite cells expansion and CSA of fast fiber types with heavy resistance training (43, 45). Moreover, contemporary studies also differ on changes to the myonuclear domain after exercise between the sexes. Although the myonuclear domains of type I and II fibers across both sexes are reportedly not altered by resistance training to volitional fatigue (43), resistance training to concentric failure is observed to increase the myonuclear domain of type II fibers in both sexes but increase the myonuclear domain of type I fibers only in adult male humans (45). Furthermore, the myonuclear domains of both type I and II fibers in moderately trained adult male humans are observed to be larger than female counterparts (28). Given the collective discrepancies in these select findings, future studies are imperative for dissecting the intersection of sex and satellite cell expansion/myonuclear accretion in mediating myofiber CSA.

In addition to the adaptive response to resistance training, the inflammatory and regenerative responses to exercise-induced skeletal muscle damage may also differ between sexes. Estrogen hormone therapy in postmenopausal female humans decreases serum creatine kinase, serum lactate dehydrogenase, and skeletal muscle mRNA expression of *IL-6*, *IL-8*, *IL-15*, and *TNF-α* in response to acute eccentric resistance exercise (167). Furthermore, male human muscles have been observed to display a greater expression of proinflammatory chemokine *CCL2* at 48 h following eccentric damage of knee extensors (168). These findings are further corroborated by reports of higher intramuscular cytokine expression of *IL-10*, *TNF-α*, and *TGF-β* in male humans at 12 and/or 24 h after eccentric knee extensions (169). These collective results therefore posit a female-specific suppression of the inflammatory and cytokine response to injurious exercise, which confers a protective effect in female muscles against exercise-induced damage. Although it is unclear whether this protective effect is mediated exclusively through E2 levels, estrogen has been proposed to attenuate muscle damage and inflammation after exercise, presumably due to its potent antioxidant capacity (189, 190). Additional studies are needed to mechanistically dissect the protective effect of estrogen against inflammation to optimize muscle recovery from exercise injury.

At the myocellular level, there are a limited number of human studies on the myogenic response to exercise-induced muscle injury (191). Moreover, the reported findings contradict on observations of sex differences. Although the eccentric phase of step exercise increased satellite cell numbers in type IIA fibers of vastus lateralis 2 and 5 days postexercise, there were no reported differences between female and male humans (192). In contrast, satellite cell expansion is observed to be greater in type I fibers of vastus lateralis in male humans 48 h following maximal eccentric knee extension, as indicated by a higher expression of PAX7+ cells than female counterparts (168). Thus, the precise role of sex on satellite cell numbers and activity during exercise injury is currently unclear and must be investigated further to account for differences in exercise mode and time points between these limited set of studies.

Although sex-inclusive research in exercise training is still limited, the recent discoveries of potential sex-dependent differences in transcriptional regulation, myocellular composition, and inflammation have important implications on the muscle adaptive response to exercise. Conceptually, these findings further illustrate the necessity for the incorporation of SABV in future exercise studies to advance our understanding of skeletal muscle adaptations and remodeling. Clinically, these sexual dimorphisms support the implementation of sex-specific exercise training and recovery protocols to best meet the distinct physiological demands of each participant, and optimize muscle health and performance accordingly.

### Sexual Dimorphisms in Skeletal Muscle Injury

In addition to exercise, muscle injuries also alter skeletal muscle structure, metabolism, and function in a sex-dependent manner. Successful muscle regeneration after injury is contingent on the myogenic differentiation of satellite cells as described earlier. At the hormonal level, several recent studies using ovariectomy in mice have reported that higher concentrations of E2 may protect female muscles against injuries induced by intramuscular cardiotoxin/barium chloride injections or freeze damage (10, 193, 194). It should be noted these findings were observed in the absence of parallel assessments in male subjects. When female and male mice are compared directly, targeted deletion of *Erβ* in satellite cells worsens muscle weight, myofiber CSA, intramuscular collagen deposition, and adipose accumulation after barium chloride injury predominantly in female muscles (11). This defective regenerative response is attributed to a role of ERβ in maintaining the muscle stem cell pool, as described earlier (11). These findings are complemented by reports of adult female mice (4–6 mo old) displaying greater efficiency than age-matched male mice in removing necrotic myofibers and a swifter restoration to baseline in CSA of centrally nucleated myofibers after cardiotoxin injury (170). Interestingly, increased intramuscular fat deposition in injured female muscles is decreased by ovariectomy, but the effect of ovariectomy performed at 12 wk of age is not reversed with concomitant E2 therapy. In male mice, intramuscular fat deposition is increased by castration performed at 12 wk of age, altogether suggesting a hormonal influence on the amount of adipocyte accumulation after muscle injury (170). In all, these findings indicate that sex hormones influence the regenerative responses to muscle injury and establish greater insights on the putative protective effect of E2.

### Sexual Dimorphisms in Muscle Disuse

Disuse muscle atrophy is induced by a combination of unloading and inactivity (195). This phenomenon can occur in clinical settings in response to joint immobilization and bed rest, or in specialized environments such as microgravity conditions during spaceflight. Regardless of setting, muscle unloading leads to loss of muscle mass due to a net loss of muscle proteins (195, 196). Consequently, disuse atrophy can be debilitating and a serious threat to overall quality of life. In humans, short-term knee immobilization leads to greater decreases in isometric and concentric strength in females than males, despite similar levels of whole muscle and myofiber atrophy between the sexes (171). The comparable levels of muscle wasting suggest muscle protein turnover does not differ between male and female humans during disuse atrophy (197). Moreover, this absence of a morphological phenotype to support the observed sex-specific functional deficits highlights a need for additional studies to elucidate the role of sex in muscle maintenance under disuse conditions.

Rodent models of hindlimb suspension/unloading are commonly utilized to study musculoskeletal disuse under microgravity conditions, with recent findings revealing contrasting sex differences in disuse atrophy. Following hindlimb suspension, the absolute loss of grip strength and the decline from baseline are greater in male rats compared with female rats (172). This finding, coupled with earlier reports that ovariectomy in adult female rats prevents muscle mass recovery after hindlimb unloading (174, 175), suggests a protective effect of the female sex hormones against disuse atrophy. Conversely, female mice have also been found to experience higher catabolic signaling and anabolic repression during the early stages of disuse atrophy after hindlimb unloading (173). Cumulatively, these findings highlight the importance of understanding how the role of sex varies across different modes of disuse atrophy and in different species.

Besides immobilization and spaceflight, disuse muscle atrophy can arise from orthopedic trauma, such as tendon tears. Recent comparisons of atrophic signaling between different models of disuse atrophy have uncovered potentially distinct molecular profiles. Although hindlimb casting (immobilization) is characterized by a classical ubiquitin-proteasome degradation signature, Achilles tendon laceration (tenotomy) is characterized by lysosomal and matrix-metalloproteinase-mediated degradation (198). Furthermore, in a surgical mouse model of rotator cuff tenotomy, a sex-dependent increase in the atrophic response corresponds with an accumulation of large autophagic vesicles solely in male muscles, revealing a novel sex-specific role for autophagy in mediating tenotomy-induced atrophy (176). Interestingly, the manner of atrophy in mice also differs between the sexes after tenotomy, with female muscles uniquely displaying a loss of myofibers, whereas myofiber size was impaired solely in male muscles (176).

## SKELETAL MUSCLE DISEASES AND TREATMENT

It is also important to elucidate the sex dimorphisms in skeletal muscle diseases and secondary pathologies, as well as in treatment results, to facilitate implementation of the most appropriate interventions in the clinical setting. The following sections highlight the effect of sex in muscular/neuromuscular disorders, associated muscle pathologies, and responses to assorted therapeutic strategies (Table 4).

**Table 4.** Summary of reported sex differences in the domains of skeletal muscle disease and treatment

Muscle Domain	Species	Female	Female and Male	Male
*Muscle/neuromuscular disorders*				
•Duchene muscular dystrophy (DMD)			
Clinical presentation (199, 200)	Human	Mild muscle weakness that presents proximally and asymmetrically		Chronic muscle degeneration/regeneration Severe muscle weakness
*mdx* mouse (201, 202)	Mouse	Attenuated loss of muscle-specific tetanic force vs. male *mdx* (sedentary) (202) ↓ Muscle regeneration and inflammation vs. male *mdx* (exercise) (201)		↓ Muscle specific tetanic force vs. female *mdx* (sedentary) (202) ↑ Regenerating fibers and inflammatory area in muscle vs. female *mdx* (exercise) (201)
•Spinal muscle atrophy (SMA)			
Prevalence (203–205)	Human			↑ Prevalence, especially in milder forms
Symptom severity (205)	Human	↑ Motor function scores in SMA types 1, 3a, and 3b vs. male patients		↑ Clinician severity scores in SMA types 2 and 3b vs. female patients
•Myasthenia gravis (MG)			
Prevalence/incidence (206, 207)	Human	↑ Prevalence of generalized MG overall and < 40 yr Earlier onset of generalized MG		↑ Prevalence of generalized MG > 50 yr
Clinical course (208–210)	Human	↓ Quality of life score, ↑ disease severity score vs. male patients (208) Greater ↓ in quality of life score with comorbidities vs. male patients (209) ↑ Likelihood of rituximab treatment (210)		↓ Quality of life score with comorbidities (209) ↑ Likelihood of low dose prednisone treatment (210)
*Secondary muscle pathologies*				
•Cancer cachexia (211)	Human	↓ Grip strength with weight loss vs. sex-matched controls		↓ Grip strength with weight loss vs. sex-matched controls ↓ Grip strength with severe weight loss vs female patients
Gastrointestinal cancer (212)	Human	↓ Strength in lower limb vs. sex-matched controls		↓ Strength and power in lower limb vs. sex-matched controls ↓ Physical function and fatigue quality of life scores vs. sex-matched controls
Colorectal cancer (213)	Mouse	↓ Body weight and muscle mass vs. sex-matched controls		↓ Strength and muscle protein in hindlimb vs. sex-matched controls
Pancreatic cancer (214)	Human, Mouse	↑ Expression of Activin inhibitors vs. male patients and male mice		↑ Muscle loss and onset of loss vs female patients and sex-matched control mice ↓ Expression of Activin inhibitors vs sex-matched control mice
*Treatment responses*				
•Intermittent glucocorticoid (215)	Mouse	↓ Intramuscular triglycerides; ↑ treadmill performance vs. sex-matched controls	↑ Muscle performance; ↑ ATP concentration vs. sex-matched controls	↑ Myofiber CSA (trend) vs. sex-matched controls
Muscle transcriptome (215)	Mouse	↑ Expression of lipid metabolism genes vs. sex-matched controls		↑ Expression of calcium handling and hypertrophic signaling genes vs. sex-matched controls
•Pharmacologic proteasome inhibition (neonatal denervation) (8, 24, 216)	Mouse	↑ Longitudinal muscle growth; ↓ Muscle contractures vs. sex-matched controls		More consistent ↑ in longitudinal muscle growth; More consistent ↓ in muscle contractures vs. sex-matched controls
•Mstn-targeted deletion (217, 218)	Mouse	No effect masseter mass with 12-wk deletion vs. sex-matched controls (217) ↑ Lean mass; no effect on fat mass with 12-mo deletion vs. sex-matched controls (218)		↑ Masseter mass with 12-wk deletion vs. sex-matched controls (217) ↑ Fat mass; no effect on lean mass with 12-mo deletion vs. sex-matched controls (218)
•Pharmacologic Mstn inhibition (ACVR2B-Fc)	Mouse			
Neonatal denervation (8)	Mouse	↑ Muscle CSA, volume, weight, protein content, and longitudinal growth; ↓ Muscle contractures vs. sex-matched controls		No treatment effect vs. sex-matched controls
Pancreatic cancer cachexia (214)	Mouse	No treatment effect on muscle and fat loss vs. sex-matched controls		Restores the loss of overall fat mass and muscle mass in gastrocnemius, quadriceps, and tibialis; ↓ atrophy-associated pathways vs. sex-matched controls
Lung cancer cachexia (219)	Mouse	↑ Muscle and fat mass, rescues weight loss, restores activity levels, ↓ mortality when co-administered with a ghrelin receptor agonist vs. sex-matched controls		No treatment effect on weight loss, activity levels, and survival when coadministered with a ghrelin receptor agonist vs. sex-matched controls
•*Mcat* overexpression (hindlimb unloading) (220)	Mouse	Attenuates losses in body weight, muscle mass of soleus and tibialis anterior, and myofiber CSA of plantaris vs. sex-matched controls		No treatment effect on body weight, muscle mass, and myofiber CSA vs. sex-matched controls
•Pharmacologic inhibition of TGF-β signaling with losartan (hindlimb unloading) (221)	Rat	Attenuates losses in muscle weight and myofiber CSA of soleus vs. sex-matched controls		No treatment effect on soleus mass and myofiber CSA vs. sex-matched controls

ACVR2B-Fc, activin receptor type 2B with an Fc tag; CSA, cross-sectional area; MSTN, myostatin; TGF-β, transforming growth factor-beta.

### Sexual Dimorphisms in Muscular and Neuromuscular Disorders

Skeletal muscle can be compromised by a variety of intrinsic muscular disorders or by disorders accompanied by neurologic dysfunction (neuromuscular disorders). Within this broad scope lies a wide spectrum of skeletal muscle deformities and dysfunctions caused by genetic mutations that primarily or secondarily impair skeletal muscle function (222). Surprisingly, many of these mutations display X-linked inheritance. X-linked recessive disorders, such as Duchenne muscular dystrophy (DMD) (223), predominantly affect males, as female carriers with a mutated X chromosome typically remain asymptomatic due to compensation by the wild-type allele. In this subsection, we will review key recent sex-specific findings in the phenotypic presentation of the following neuromuscular disorders: DMD, spinal muscle atrophy (SMA), and myasthenia gravis (MG).

Duchene muscular dystrophy (DMD) is one of the most frequent lethal inherited diseases, affecting ∼1:3,500 to 5,000 live male births compared with 1:50,000,000 live female births (199, 223, 224). This form of muscular dystrophy is caused by a deficiency in the dystrophin protein which, in turn, promotes fragility of the myofiber. As the myofiber becomes more unstable, there is a higher likelihood of muscle injury or necrosis (201). These changes in muscle mechanical properties are mostly studied using *mdx* mice as a model for DMD (225). Although *mdx* mice have a point mutation that prevents expression of functional dystrophin, they exhibit minimal clinical symptoms and lifespan reduction compared with the human phenotype. The translatability of this model is further confounded by sex, which impacts the phenotype of *mdx* mice in several ways. First, the contractile and passive mechanical properties of skeletal muscle in *mdx* mice differ between sexes, with younger female *mdx* mice displaying higher specific tetanic force of their extensor digitorum longus muscles, and older female *mdx* mice experiencing stiffer extensor digitorum longus muscles compared with their aged-matched male counterparts under sedentary conditions (202). Second, the regenerative response to exercise in *mdx* mice differs between sexes. Following treadmill running, diaphragm muscles of female *mdx* mice display fewer centrally nucleated myofibers and a reduced presence of inflammatory infiltrates than those of male mice, presumably due to the role of estrogen in mitigating exercise-induced increases in inflammation and injury (201). These key differences in mechanical properties and regenerative response may potentially account for the milder muscle weakness observed in rare female patients with DMD, which typically presents proximally and asymmetrically (199, 200). In all, the sex dimorphisms observed in *mdx* mice highlight the necessity to consider sex when using this model of DMD for biomedical research.

Spinal muscle atrophy (SMA) is an autosomal recessive neuromuscular disorder that is caused by homozygous deletions and point mutations in the survival motor neuron 1 gene (*SMN1*) (223). There are three subtypes of SMA that differ by achieved motor milestones (226). SMA type 1 is the most severe form with onset of symptoms less than 6 mo of age and characterized by the inability to sit without assistance. SMA type 2 is the intermediate form with symptoms seen by 6–18 mo of age and characterized by the ability to sit without aid accompanied by the inability to stand or walk alone. SMA type 3 is the mildest form with symptoms starting at 18 mo of age or older with preserved ability to stand and walk alone. The clinical severity of SMA is putatively modulated by two known genetic factors, neuronal apoptosis inhibitory protein gene (*NAIP*) and survival motor neuron 2 gene (*SMN2*) (203). Among Japanese patients, SMA type 3 is more prevalent in male patients without *NAIP* deletion and with a high *SMN2* copy number (203). This finding suggests that sex-related modifiers in *NAIP* expression and *SMN2* copy numbers ultimately contribute to SMA disease severity, and supports prior reports of male predominance in milder forms of SMA (204). The effects of these sex dimorphisms in patients with SMA are corroborated by a recent study of two of the largest human SMA registry databases in the world, which reported that more male family members are affected by SMA than female family members (205). Besides prevalence, analyses from these databases revealed putative sex differences in symptom severity. Specifically, male patients with SMA types 2 and 3b exhibited more severe clinical symptoms, whereas female patients with SMA types 1, 3a, and 3b displayed greater motor function, than respective sex-matched counterparts (205). However, older studies have reported no sex differences in sex ratio or symptom severity in SMA types 1 or 2 (203, 227, 228). Thus, more investigation is needed to elucidate the influence of sex dimorphisms on both SMA pathophysiology and its clinical course.

The presentation of myasthenia gravis (MG) also differs between male and female patients. In this neuromuscular autoimmune disease, antibodies target the acetylcholine receptors at the neuromuscular junction, causing skeletal muscle weakness and fatigue (223). This weakness affects skeletal muscles throughout the body, including those in the eyes, airway, arms, and legs (229). Among patients with MG, the prevalence and onset of generalized MG is 11% higher and 10 years earlier in females than males, respectively (206). Despite the higher overall incidence of generalized MG in female patients, this form of MG is 30% more prevalent in female patients under the age of 40, but 25% higher in male patients above the age of 50 (206). Thus, the incidence of generalized MG is dependent on both sex and age. The female preponderance of this disease may be due in part to skewed X chromosome inactivation among young females (207), which supports the higher incidence of autoimmunity in individuals with two X chromosomes (230). In addition to the sex-specific prevalence of generalized MG, female patients often have a more compromised health-related quality of life, as indicated by decreased quality of life and harsher disease severity scores compared with male patients (208). Among patients with MG living in China, female patients experience a faster decline in quality of life as the number of comorbidities increase (209). Importantly, female patients with MG are also less likely to be treated with low-dose prednisone and more likely to be treated with rituximab than males (210). Thus, potential sex dimorphisms in clinical outcomes of MG may be partially attributed to treatment discrepancies between male and female patients.

### Sexual Dimorphisms in Secondary Skeletal Muscle Pathologies

In humans, skeletal muscle comprises ∼40–50% of the total body mass (1). Given its functionality and abundance, it is not surprising that secondary muscle pathologies arise in many congenital and acquired diseases. This subsection will specifically focus on sex dimorphisms associated with muscle wasting stemming from cachexia.

Cachexia, also referred to as wasting syndrome, is a metabolic disorder involving the progressive loss of skeletal muscle mass because of an underlying chronic illness, such as cancer (213). Due to the underlying pathologies, cachexia is not limited to skeletal muscle. Instead, it is a multifactorial syndrome that results in accelerated weight loss and sustained catabolism that is irreversible with nutritional therapies (231, 232). In cancer cachexia, secondary muscle wasting affects ∼60–80% of patients with advanced tumors (233). As cancer cachexia leads to progressive decline in skeletal muscle mass, patients also suffer from inflammation, loss of muscle function, and fatigue (234). A hallmark of cachexia is reduced muscle strength (211). Here, sex has been shown to impact muscle function in both upper and lower extremities of patients with cachexia. Hospitalized male patients with cachexia under the age of 70 with severe weight loss have a greater reduction in hand grip strength compared with female patients (211). When compared with sex-matched healthy controls, male patients with gastrointestinal cancer cachexia display decreased quadriceps strength and lower limb power, whereas female patients only exhibit decreased quadriceps strength (212). Male patients also experience reduced physical function and increased fatigue quality of scores in their lower limbs, whereas female patients do not (212).

Various animal models that induce tumor formation have also been used to study cancer cachexia. In mice, synergistic mutation of the *Apc* gene and *Pik3ca* results in the development of aggressive intestinal cancer (235) and is associated with pronounced sex differences. When compared with sex-matched controls, female tumor bearing mice are smaller in body weight and have reduced skeletal muscle mass whereas male tumor bearing mice experience greater reductions in extensor digitorum longus muscle protein and specific force (213). However, these mice lack certain aspects of the syndrome documented in human patients (213), which lessens the translational fidelity of this mouse model. Care must be taken to distinguish whether observed sex differences in cancer cachexia are due, in part, to the animal model studied, or to cancer-related fluctuations in hormone levels. In contrast, mice and human patients with pancreatic cancer cachexia share similar sex dimorphic characteristics in progressive wasting and muscle expression of Activin A, which has been previously identified as a cachectic factor due to its role in inducing lean muscle loss and inhibiting adipogenesis (121, 236). Specifically, male mice experience an earlier onset of muscle wasting than females, whereas female mice express higher levels of Activin inhibitors in the muscle during early cachexia (214). In comparison, male human patients experience a greater and faster loss in both the quantity and percentage of skeletal muscle than female patients, whereas female patients display greater muscle expression of endogenous Activin family ligand inhibitors (214). Thus, these corresponding murine and human studies further reveal that in pancreatic cancer cachexia, Activin is associated with wasting in male muscles (214). Critically, the limited number of studies documenting potential sex differences in cachexia-induced muscle wasting underscores the importance of performing rodent and human models in parallel to improve the translatability of such findings. In all, emerging sex-inclusive studies on different forms of cancer cachexia have consistently revealed more severe phenotypes in male patients for both muscle wasting and diminished muscle function.

### Sexual Dimorphisms in Skeletal Muscle Treatment

The following section will explore the role of sex in assorted therapies for improving muscle health and performance. Although glucocorticoid steroids are commonly prescribed for many inflammatory conditions, including muscular dystrophy and myasthenia gravis, shorter regimens have recently been shown to increase muscle-specific force and ATP concentrations in both male and female mice (215). However, the pathways by which this therapeutic benefit is conferred differ between the sexes. Weekly prednisone treatment upregulates myofiber expression of genes governing calcium handling and hypertrophic signaling in male mice, leading to modest increases in myofiber CSA (215). In comparison, genes governing lipid metabolism are upregulated in female mice with weekly prednisone exposure, leading to reduced intramuscular triglycerides and increased endurance (215). These transcriptomic changes further correspond to a sex-specific glucocorticoid-receptor DNA-binding pattern. Thus, weekly glucocorticoid enhances muscle performance through distinct sex-dependent pathways.

Besides the primary muscular/neuromuscular disorders described earlier, muscle contractures arising secondarily from cerebral palsy, neonatal brachial plexus injury (NBPI), and assorted neurologic disorders dramatically reduce joint range of motion and limit the functional use of affected limbs for activities of daily living. These contractures are caused by impaired longitudinal growth of denervated muscles (237, 238), which is governed by elevated levels of proteasome-mediated muscle protein degradation (24). As proof of concept, pharmacologic treatment with bortezomib, a 20S proteasome inhibitor, restores longitudinal muscle growth and prevents forelimb contractures in a surgical mouse model of NBPI (24, 216). Furthermore, bortezomib reduces contracture severity more consistently in male mice over the course of 4, 8, and 12 wk (8, 216). Although bortezomib improves longitudinal muscle growth in both sexes at 4 and 12 wk of treatment, muscle length is preserved only in bortezomib-treated male mice at 8 wk, whereas proteasome activity is reduced at 8 and 12 wk of treatment in male mice alone (8, 216). These findings highlight the subtle sex differences in response to systemic proteasome inhibition. Since bortezomib and other available proteasome inhibitors are associated with cumulative toxicity and cannot be safely translated to patients, muscle-specific regulators of protein balance were also targeted to restore the dysregulation in proteostasis during contracture development. Here, pharmacologic inhibition of MSTN signaling with the soluble decoy receptor, ACVR2B-Fc, attenuates proteasome activity, reduces atrophy, improves deficits in longitudinal growth, and reduces contracture severity, but only in the denervated muscles of female mice at 4 wk post-NBPI (8). These latter findings also extend earlier reports of an inability of ACVR2B-Fc treatment to prevent atrophy in male muscles in a juvenile mouse model of sciatic nerve resection (239), and posit a potential sex-specific role for MSTN signaling in mediating neonatal denervation atrophy.

Sex discrepancies in MSTN-targeted therapies have garnered more recognition in recent years (25). The sex-divergent response to the ACVR2B-Fc decoy receptor may be partially impacted by the condition for which it is used. In a mouse model of early pancreatic cancer cachexia, ACVR2B-Fc treatment preserves skeletal muscle loss in assorted hindlimb muscles and fat loss only in male mice (214). When coadministered with anamorelin, a ghrelin receptor agonist, ACVR2B-Fc increases skeletal muscle and fat mass, restores spontaneous activity, and reduces overall mortality only in female mice with lung cancer cachexia (219). This sex-specific response is dependent on ovarian function, as the therapeutic benefits associated with the combination therapy are lost in ovariectomized mice (219). Similarly, although targeted muscle deletion of *Mstn* during skeletal maturity increases masseter mass solely in male mice (217), its long-term deletion preferentially increases lean muscle mass in aged female mice and fat mass in male mice (218). One putative mechanism for these discrepancies may lie with sex-related differences in the temporal expression of MSTN in skeletal muscles (8). Compared with male counterparts, adult female mice display an increased transcriptional and translational expression of latent Mstn in their hindlimb muscles (240), and female humans tend to have higher gene expression of the *ACVR2B* receptor (31). Thus, these sex-intrinsic differences in MSTN and receptor gene expression may account for the sex-specific responses to MSTN-targeted therapies described here, and may also underlie the mixed clinical outcomes for MSTN inhibitors in treating DMD and other muscle disorders (25, 241).

Finally, emerging research on therapies for mitigating disuse atrophy has also identified sex-specific treatment responses. Intriguingly, successful restoration of muscle mass stemming from these treatments has been observed primarily in female muscles. Global overexpression of mitochondria-targeted catalase (*Mcat*) diminishes the effects of hindlimb unloading-induced atrophy in soleus, tibialis anterior, and plantaris muscles, and is associated with reduced mitochondrial oxidative stress only in unloaded muscles of female mice (220). However, it remains to be elucidated whether the observed sex-specific protections against disuse atrophy can be wholly attributed to alterations in mitochondrial dynamics. Similarly, administration of losartan, an angiotensin II type 1 receptor blocker that inhibits canonical TGF-β signaling, attenuates losses in soleus muscle weight and myofiber CSA after hindlimb unloading, but only in female rats (221). This sex-dependent protective effect against disuse atrophy is associated with a reduction in Smad2/3 phosphorylation, indicating that losartan alters atrophic outcomes in female muscles by preventing canonical TGF-β signaling (221). In all, these sex-specific treatment outcomes further illustrate the complex relationship between sex and muscle deconditioning during disuse.

## LIMITATIONS

Our review has several limitations. First, the findings of sex dimorphisms from animal studies described in this review have primarily been observed in one species. As such, it is possible that these sex dimorphisms are species-specific. Therefore, future studies are needed to replicate these experiments across multiple species to validate the findings. Second, we have limited the scope of our review to muscle development, homeostasis, adaptive responses, and selected pathologies. It is entirely possible that we have excluded relevant findings of sex dimorphisms in other domains of skeletal muscle. Finally, we intend for this review to be an overview of recent advances, rather than an exhaustive list of sex differences in muscle research. As we have primarily limited our discussion of pertinent findings to the past 15 years, our review will undoubtedly omit key earlier findings.

## CONCLUSIONS

Sex differences appear throughout skeletal muscle development, homeostasis, adaptive responses, and diseases, deepening our understanding of these processes. Sex dimorphisms in skeletal muscle are present at all stages of development, beginning as early as the embryonic period and continuing through the life span. At the myocellular and hormonal levels, sex discrepancies between females and males govern the maintenance of skeletal muscle homeostasis. Satellite cell-mediated muscle regeneration and adaptive responses to exercise and injury that occur are also regulated in a sex-dependent manner. These sex-specific biological and physiological distinctions complement the wide range of sex dimorphisms discovered in skeletal muscle disorders, secondary pathologies, and therapeutic interventions for such diseases. Given the prevalence of sex-based differences in skeletal muscle, there is a need for future research to include both female and male subjects to advance our knowledge of muscle biology. If possible, researchers should revisit their prior findings and reanalyze their results according to sex so that no sex dimorphism goes unnoticed. Not only does the consideration of SABV in skeletal muscle research enhance scientific rigor, but it also increases the likelihood of improving healthcare outcomes for all patients.

## GRANTS

This work was supported by grants from the NIH R01HD098280-01 (to R.C.), as well as funding from the Cincinnati Children’s Hospital Division of Orthopaedic Surgery and Junior Cooperative Society (to R.C.).

## DISCLAIMERS

The respective funding sources were not involved in the study design; in the collection, analysis, and interpretation of data; in the writing of the report; and in the decision to submit the paper for publication. The content is solely the authors’ responsibility and does not necessarily represent the official views of Cincinnati Children’s Hospital Medical Center.

## DISCLOSURES

No conflicts of interest, financial or otherwise, are declared by the authors.

## AUTHOR CONTRIBUTIONS

M.E.E., A.S.E., and Q.G. conceived and designed research; M.E.E. and A.S.E. analyzed data; M.E.E. and A.S.E. interpreted results of experiments; M.E.E. and A.S.E. prepared figures; M.E.E. and A.S.E. drafted manuscript; M.E.E., A.S.E., Q.G., and R.C. edited and revised manuscript; Q.G. and R.C supervised the review; M.E.E., A.S.E., Q.G., and R.C. approved final version of manuscript.
